# Predictive factors for successful non-operative treatment and achieving MCID improvement in health-related quality of life in adult spinal deformity

**DOI:** 10.1186/s12891-022-05757-0

**Published:** 2022-08-22

**Authors:** Jason Pui Yin Cheung, Hei Lung Wong, Prudence Wing Hang Cheung

**Affiliations:** Department of Orthopaedics and Traumatology, The University of Hong Kong, 5th Floor, Professorial Block, 102 Pokfulam Road, Pokfulam, Hong Kong SAR, China

**Keywords:** Adult spinal deformity, Conservative treatment, ODI, SRS-22r, EQ-5D, MCID

## Abstract

**Background:**

Adult spinal deformity is a spectrum of degenerative spinal diseases with increasing prevalence and healthcare burden worldwide. Identification of patients who are more likely to improve through conservative management may reduce cost and potentially prevent surgery and its associated costs and complications. This study aims to identify predictive factors for MCID in improvement of ODI and SRS-22r questionnaires in patients with adult spinal deformity treated with conservative treatment.

**Methods:**

A prospective, observational cohort study of 46 patients was conducted at a spine specialist clinic. Inclusion criteria were 30–80 years of age, diagnosis of neglected adolescent idiopathic scoliosis, de-novo scoliosis, degenerative spondylolisthesis, and sagittal plane deformities (thoracic hypokyphosis, lumbar hypolordosis), presenting with mechanical back pain with or without radicular leg pain. All patients received conservative management including medication and physiotherapy. Radiological and clinical parameters were measured at baseline and at 1-year follow-up. Primary outcomes were ODI and SRS-22r scores. Secondary outcomes were EQ-5D-5L scores and requiring spine surgery during conservative treatment. Predictors for MCID improvement in ODI and SRS-22r were identified using multivariate regressions and receiver operating characteristic (ROC) analyses.

**Results:**

At baseline, patients who reached MCID in ODI and/or SRS-22r showed less comorbidities (diabetes mellitus, hypertension, ischemic heart disease, osteoarthritis, cancer), smaller range of lateral spinal flexion, larger trunk shift, larger pelvic incidence, a higher EQ-5D-5L anxiety/depression dimension score, a lower SRS-22r total score, and presence of spondylolisthesis. Lateral flexion range < 25 degrees, trunk shift > 14 mm, pelvic incidence > 50 degrees, EQ-5D-5L anxiety/depression dimension score > 1, and SRS-22r total score < 3.5 were the cut-off values generated by ROC analysis.

**Conclusions:**

Both radiological and clinical predictive factors for MCID improvement in health-related quality of life were identified. Future research should identify subgroups of patients who are responsive to specific conservative treatment modalities, so as to provide information for personalized medicine.

**Level of evidence:**

II

## Background

Due to the growing number of patients with back pain and deformity, there is an increased interest in improving our knowledge of its pathogenesis, of optimal corrective surgeries and on the impact on health-related quality of life (HRQoL). However, despite our improved knowledge of adult deformity pertaining to surgical techniques and outcomes, our current understanding of the role of conservative treatment is very limited. This is an important treatment modality for degenerative conditions that is often overlooked. Coupled with the high risk of complications up to 80% with a reoperation rate of 50% [1, 2], conservative/nonoperative management should be carefully dissected to provide clinicians with better guidelines before administering any invasive interventions. This is especially important when taking into account the high cost of these surgeries [3].

Several studies comparing surgical and nonoperative outcomes have been published in the Caucasian population [4–8]. However, there is selection bias as none provided a standardized protocol or definition of conservative treatment according to the therapy given and its failure [9]. Most available evidence on nonoperative outcomes is also retrospective [10], and lacks consensus for adult spinal deformity. The armamentarium of conservative management options is also variable with bracing, manipulation, physical therapy and epidural injections [10]. Thus, the current evidence is limited and results are difficult to replicate [4]. In addition, no study has been performed in the Asia-Pacific population. There are obvious ethnic and cultural variations that influence the outcomes to specific management options for adult spinal deformity [11–13].

It is likely that a large population of adult spinal deformity patients do not require surgery and may benefit from a conservative approach, or at least delay the surgery until patient medical status or comorbidities are first optimized. By determining the patient profile that responds well to conservative treatment, we can identify those patients who can avoid surgery. This is particularly important for risk calculation as surgery for adult deformity is considered high risk [14]. As such, identification of the factors that predict successful non-operative treatment in adult deformity is necessary. This can deliver a patient profile most likely to avoid surgery with conservative treatment, thereby allowing us to prescribe the best individualized treatment protocol via an evidenced-based approach. Clarification of conservative treatment protocols and factors contributing to successful non-operative treatment is necessary. Thus, this study aims to determine the predictive factors for successful non-operative treatment for adult spinal deformity as identified by improved quality of life measures beyond the minimal clinically improved difference (MCID).

## Methods

### Study population

We prospectively recruited patients aged 30–80 with adult spinal deformities from December 2017 to December 2019. Patients with diagnosis of adult spinal deformity including neglected adolescent idiopathic scoliosis, de-novo scoliosis, degenerative spondylolisthesis, and sagittal plane deformities (thoracic hypokyphosis, lumbar hypolordosis) were included. All patients presented with mechanical back pain with or without leg pain. Patients recruited must also be candidates for conservative treatment determined by orthopaedic specialists during consultation. Exclusion criteria included the diagnosis of spondyloarthritis, or if the deformity was a result of neuromuscular causes, trauma, infections and or tumors. Patients who were not suitable for conservative treatment, such as those with lower limb motor deficit and allergy to non-steroidal anti-inflammatory drugs were also excluded. Ethics approval was obtained from the local Institutional Review Board.

There were 46 patients recruited of 120 patients suitable in the study period. The other 74 patients had neurological deficit and were thus not included in the study. Of the remaining 46 patients included in the study, 3 patients failed conservative treatment and switched to surgery during the study duration and 5 patients defaulted final follow-up. A total of 38 patients remained for study analysis.

### Conservative treatment regimen

Study participants received a standard conservative care including active physiotherapy (such as back strengthening and mobilization, hydrotherapy, passive leg stretching if radicular symptoms present), or passive treatment (like heat, traction, massage or bracing) if patients were not tolerating, and when deemed necessary the prescription of analgesics, facet or epidural injections according to standard practices for 1 year since baseline. The physiotherapy was provided to patients by the same trained physiotherapist twice weekly for 3 months then weekly thereafter up till 6 months before self-exercises only were provided with monthly checkups. Analgesics provided included paracetamol 500 mg 4 times daily as required, non-steroidal anti-inflammatory agents (naproxen 250 mg twice daily as required or diclofenac slow release 100 mg daily as required) and pregabalin 75 mg twice daily for radicular symptoms. If there was no improvement in radicular symptoms at 3 months with the physiotherapy and pregabalin, the patient would be offered an epidural injection. Facet injections would also be offered for patients with predominantly back-extension related pain if no improvement was reported with the prior mentioned conservative treatment. There were no deviations to this protocol for our subjects.

### Study measures

i) *Clinical parameters*

Clinical and radiographic parameters were collected at baseline recruitment and at 1-year follow-up. Clinical data included demographics and questionnaires administered via face-to-face interviews. Patient demographics included age, gender, ethnicity, education, employment status, body height and weight, BMI, smoking status, location of pain, duration of illness since onset, presence of comorbidities (diabetes mellitus type I and II, hypertension, ischemic heart disease, osteoarthritis, cancer), Charlson Comorbidity index, use of walking aids, back range of motion (flexion, extension, lateral flexion) and timed up and go (TUG) test (measuring time needed for patient to rise from an arm chair, walk 3 m, turn, walk back, and sit down again) [15]. Results were recorded to assess patient mobility. Prescriptions of physiotherapy and/or steroid injection were also recorded.

ii) *Patient-reported outcomes*

Questionnaires under study included the Oswestry Disability Index (ODI), the refined 22-item Scoliosis Research Society (SRS-22r) questionnaire and the EuroQol 5-dimension 5-level (EQ-5D-5L) questionnaire for assessing disability and HRQoL. ODI is one of the principal outcome measures used in the management of spinal disorders [16]. It is scaled from 0 to 100, with larger value showing more severe disability. The normal population has a mean score of 10.19 (SD range of 2.2–12.0), while the population with primary back pain has a mean score of 27 (SD range of 5.8–23.6). The index can be aggregated into 5 levels (0–20%, 21–40%, 41–60%, 61–80%, and 81–100%), each indicating minimal, moderate, severe, crippling and bed-bound disability respectively [16, 17]. SRS-22r is a disease-specific tool for evaluating HRQoL outcomes of treatment for scoliosis patients. It is divided into five domains: pain, function, self-image, mental health and satisfaction with treatment, each scoring from 1.0 to 5.0, and total score as average of all domains. Higher value indicates better HRQoL in the corresponding domain [18, 19]. EQ-5D-5L is a generic health survey used to measure changes in HRQoL and to compare improvement across different interventions. This tool consists of five dimensions of health — mobility, self-care, usual activities, pain/discomfort, and anxiety/depression, with the respondent choosing from 5 levels of responses defining problem in each dimension: no, slight, moderate, severe, extreme problems. The responses of the dimensions are then coded into a five-digit code which describes the health state of the respondent as per EQ-5D-5L user instruction. These health states were than scaled to the population’s EQ-5D value set via cross-walking [20–22]. The total EQ-5D-5L index was found as a utility score whereby 1 and 0 represents instrument-defined full health and death respectively [23].

iii) *Radiological parameters*

Standing posteroanterior and lateral spinal whole spine and focal lumbosacral spine radiographs were taken at baseline and at 12 months. Coronal parameters included the major curve Cobb angle, any lateral listhesis, C7-Central Sacral Vertical Line (CSVL) deviation, and trunk shift. Sagittal parameters included the T5-T12 kyphotic angle, L1-S1 lordotic angle, pelvic tilt, sacral slope, pelvic incidence and sagittal vertical axis (SVA) deviation. The presence of spondylolisthesis and the level involved were noted with its degree of slip, slip angle, slip distance, and disc height [24–26].

### Statistical analysis

Descriptive statistics was used to characterize the study sample with means and standard deviations for continuous variables, and frequencies in percentages for categorical variables. The primary outcome measure was successful treatment, defined as one MCID improvement in ODI or SRS-22r. The MCIDs for ODI and SRS-22r for spinal deformity patients with non-surgical treatments are 2.45 and 0.11 respectively [27]. Secondary outcome measures include the number or count of patients that required surgery.

Changes in parameters pre- and post-treatment were detected using paired t-test. Pearson’s correlation tests were performed between the three HRQoL measures (ODI, SRS-22r, EQ-5D-5L). Patients who achieved and did not achieve MCID were compared in terms of baseline radiological and HRQOL parameters. For data with skewed distribution, non-parametric tests were used. Receiver Operating Characteristic (ROC) analysis was used to determine the cut-off value for quantitative variables using the Closest-To-(0,1) method [28]. Univariate analysis was conducted to identify possible associated factors. Factors with a *p*-value < 0.20 in univariate analysis were then included in the stepwise multivariate logistic regression to develop the prediction model. Numerical predictors were further analyzed using ROC analysis. Interrater and intrarater reliability were assessed using intraclass correlation coefficient, which was > 0.70 for all parameters measured, representing satisfactory reliability. All data were analyzed using SPSS 26.0 (IBM, USA). A *p*-value of < 0.05 was considered statistically significant.

## Results

**Fig. 1:**
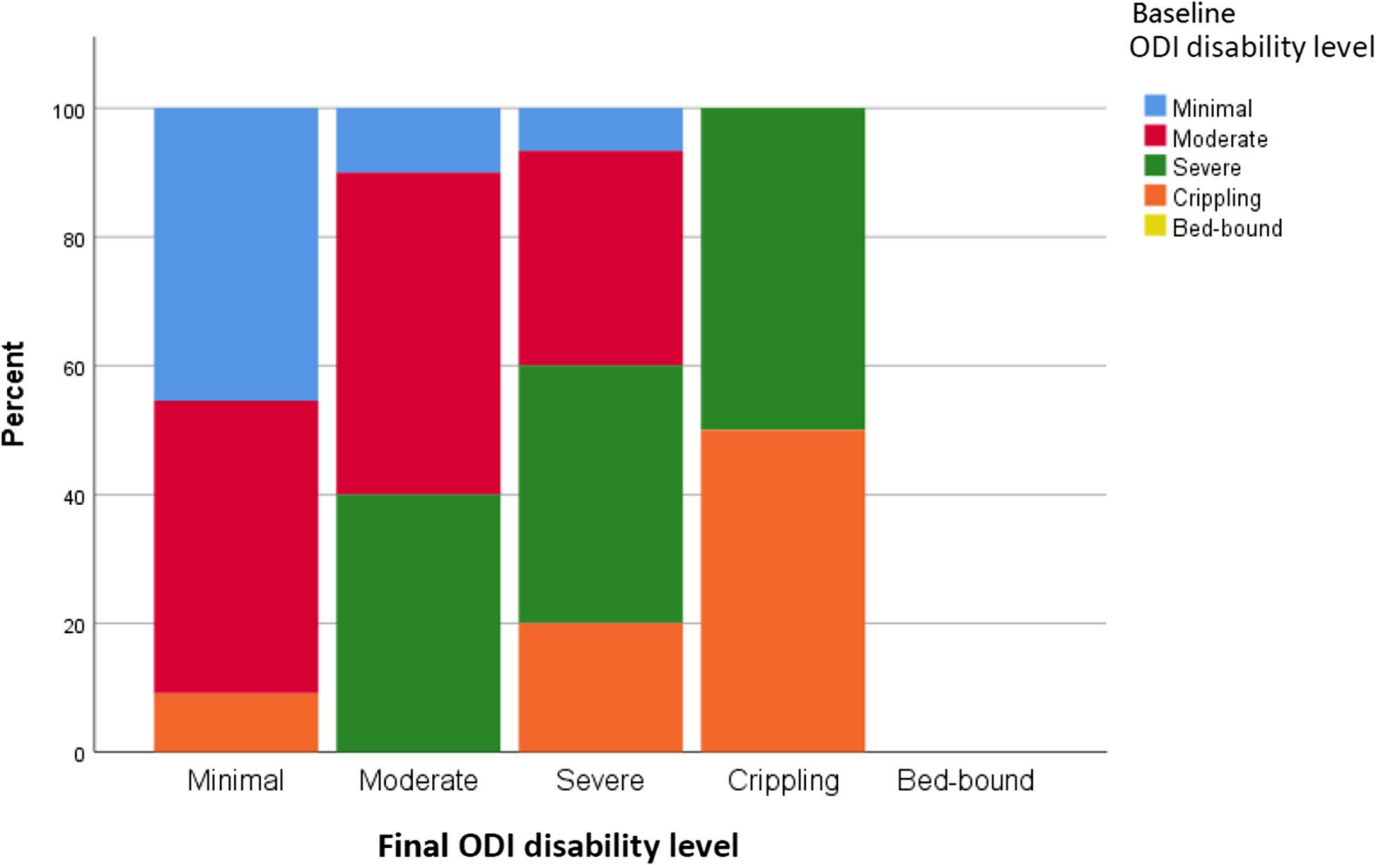
Bar chart showing frequencies of final ODI disability level by baseline ODI disability level

Patients (80% women) had a mean age of 64.3 ± 9.8 years, height of 154.0 ± 9.2 cm, and BMI of 24.4 ± 4.4 kg/m^2^. During the study period, 3 patients switched to surgical treatment. Baseline ODI and SRS-22r scores showed moderate levels of disability and severity of clinical symptoms respectively (Table 1). In the overall cohort, there were no significant changes in any of the three HRQOLs from baseline to 1-year follow-up (Fig. 1). At both baseline and 1 year, all three HRQOLs (ODI, SRS-22r and EQ-5D-5L) showed significant correlation with each other (*p* <  0.001) (Table 2). Moreover, no significant radiological change was seen in any of the aforementioned spinopelvic parameters. After removing the subjects with no change in ODI and comparing only the 21 who improved versus the 15 who deteriorated for the ODI score change, we found a mean difference of 23.6 (95% CI 17.1–30.2) between groups. Similarly for SRS-22r, when comparing only the 19 improved versus the 18 deteriorated subjects, the mean difference was 0.863 (95% CI 0.632–1.094) between groups. However, no differences were observed for radiological parameters between these two groups.

**Table 1:** Paired sample t-test between patient outcome scores at baseline and final assessment

Patient outcome measures	Initial score	Final score	Changes of scores	*p*-value
	Mean (SD)			
ODI	32.8 (17.4)	36.6 (17.3)	3.86 (14.68)	0.113
SRS-22r				
Domain scores				
Function	3.74 (0.63)	3.87 (0.68)	0.13 (0.77)	0.309
Pain	3.37 (0.83)	3.42 (0.67)	0.04 (0.78)	0.740
Self-image	3.17 (0.64)	3.03 (0.71)	−0.14 (0.74)	0.263
Mental health	3.76 (0.78)	3.57 (0.89)	−0.19 (1.02)	0.247
Satisfaction with treatment	2.28 (0.70)	2.33 (1.02)	0.05 (1.42)	0.941
Total score	3.52 (0.52)	3.49 (0.54)	−0.03 (0.55)	0.707
EQ-5D				
EQ-5D-5L	0.637 (0.266)	0.679 (0.293)	0.042 (0.778)	0.467
EQ-VAS	64.2 (18.0)	61.6 (19.4)	−2.5 (23.5)	0.622

*ODI* Oswestry Disability Index, *SRS-22r* Refined Scoliosis Research Society 22-item, *VAS* Visual analogue scale

**Table 2:** Correlation analysis between the three baseline health-related quality of life outcome scores and between initial and final scores

**Patient outcome measures**	**Correlation between initial and final scores**	***p*-value**
	**Pearson correlation coefficient**	
ODI	0.641	<0.001*
SRS-22r		
Domain scores		
Function	0.306	0.061
Pain	0.475	0.003*
Self-image	0.401	0.013*
Mental health	0.257	0.119
Satisfaction with treatment	−0.376	0.084
Total score	0.472	0.003*
EQ-5D		
EQ-5D-5L	0.219	0.192
EQ-VAS	0.203	0.228
**Between outcome measures**	**r**	***p*-value**
ODI and SRS-22r total scores	0.788	< 0.001*
ODI and EQ-5D-5L	0.748	< 0.001*
SRS-22r total score and EQ-5D-5L	0.764	< 0.001*

* statistical significance at *p* < 0.05

*ODI* Oswestry Disability Index, *SRS-22r* Refined Scoliosis Research Society 22-item, *EQ-5D-5L* EuroQol 5-dimension 5-level, *VAS* Visual analogue scale

Within the cohort, 30.4% (*N* = 14) of patients achieved > 1 MCID improvement in ODI, while 54.3% (*N* = 25) failed to do so. 37.0% (*N* = 17) reached MCID in SRS-22r total score, while 47.8% missed MCID in SRS-22r. Among those who achieved MCID in ODI, 9 of them reached MCID in SRS-22r at the same time.

For ODI, no statistically significant difference in baseline radiological and HRQOL parameters was found between the patients who achieved and those who did not achieve MCID. Only absence of comorbidities (*p* = 0.014) and smaller range of lateral spinal flexion (*p* = 0.011) were identified as significant predictors for achieving MCID in univariate analysis and close to statistical significance in the multivariate regression model (*p* = 0.054 and *p* = 0.061 respectively) (Table 3) for reaching MCID in ODI. ROC analysis found a lateral flexion range cut-off of < 25 degrees (*p* = 0.005) achieved MCID (Table 4). For SRS-22r, whether patients reached or not reached MCID was significantly correlated with each of the following factors: trunk shift, pelvic incidence, number of cases of neglected AIS, range of lateral spinal flexion, self-rated back pain, EQ-5D-5L pain and anxiety/depression dimension scores, SRS-22r pain, and SRS-22r total score (Table 3). The following cut-off values were defined: trunk shift > 14 mm, pelvic incidence > 50 degrees, lateral flexion range < 25 degrees, EQ-5D-5L anxiety/depression dimension score > 1, and SRS-22r total score < 3.5 and these were significant predictors reaching MCID in SRS-22r score (all at *p* <  0.05) (Table 4).

**Table 3:** Univariate and multivariate analysis for predictors of MCID improvement in ODI and/or SRS-22r scores

Factors	Univariate analysis		Multivariate analysis	
	Pearson’s correlation coefficient	*p*-value	Odds ratio for successful treatment (95% CI)	*p*-value
**Outcomes - ODI MCID** Multivariate regression statistics: *X*^2^ = 10.878 (*p* = 0.004), −2 Log likelihood = 36.214, Cox & Snell R Square = 0.261, Nagelkerke R^2^ = 0.357				
Presence of comorbidities	−0.396	0.014	0.18 (0.03–1.03)	0.054
Range of lateral spinal flexion	−0.417	0.011	0.92 (0.85–1.00)	0.061
**Outcomes - SRS-22r MCID** Multivariate regression statistics: *X*^2^ = 14.506 (*p* = 0.013), −2 Log likelihood = 23.737, Cox & Snell R Square = 0.404, Nagelkerke R^2^ = 0.543				
Trunk shift	0.407	0.028	1.07 (0.96–1.19)	0.204
Pelvic incidence	0.422	0.009	1.05 (0.96–1.15)	0.322
Lateral flexion range	−0.592	< 0.001	0.91 (0.81–1.03)	0.138
EQ-5D-5L anxiety/decompression dimension score	0.361	0.026	2.21 (0.19–25.70)	0.528
SRS-22r total score	−0.435	0.006	0.15 (0.002–10.03)	0.567
**Outcomes - Both ODI and SRS-22r MCIDs** Multivariate regression statistics: *X*^2^ = 22.346 (*p* < 0.001), −2 Log likelihood = 15.282, Cox & Snell R Square = 0.472, Nagelkerke R^2^ = 0.716				
Presence of comorbidities	−0.382	0.018	0.11 (0.01–1.95)	0.131
Lateral flexion range	−0.400	0.016	0.85 (0.71–1.02)	0.073
Presence of spondylolisthesis	0.439	0.007	15.70 (0.72–342.67)	0.080
Physiotherapy prescription	−0.310	0.059	0.01 (< 0.001–0.89)	0.044

*MCID* Minimal clinically important difference, *CI* Confidence interval, *X*^2^ Chi-square, *ODI* Oswestry Disability Index, *SRS-22r* Refined Scoliosis Research Society 22-item, *EQ-5D-5L* EuroQol 5-Dimension 5-Level

**Table 4:** Receiver operating characteristics analysis and cut-off values for the prediction of outcomes of MCID improvement of ODI, SRS-22r scores and both ODI and SRS-22r scores

Parameters	AUC	*p*-value	Cut-off value	Sensitivity (%)	Specificity (%)
ODI MCID					
Range of lateral spinal flexion (degrees)	0.739	0.005*	25.0	69.2	56.5
SRS-22r MCID					
Trunk shift (mm)	0.711	0.049*	14.0	66.7	82.4
Pelvic incidence (degrees)	0.720	0.009*	50.0	66.7	59.1
Lateral flexion range (degrees)	0.841	< 0.001*	25.0	81.3	70.1
EQ-5D-5L anxiety/decompression dimension score	0.697	0.028*	1.0	56.3	81.8
SRS-22r total score	0.743	0.002*	3.5	81.3	63.6
Both ODI and SRS-22r MCIDs					
Lateral flexion range (degrees)	0.777	0.018*	25.0	75.0	53.6

*AUC* Area under the curve, *ODI* Oswestry Disability Index, *SRS-22r* Refined Scoliosis Research Society 22-item, *EQ-5D-5L* EuroQol 5-Dimension 5-Level

For patients who reached MCID in both ODI and SRS-22r, significant predictors in univariate analysis included absence of comorbidity, small range of lateral spinal flexion, presence of spondylolisthesis, and absence of physiotherapy prescription (Table 3). Multivariate regression showed that smaller range of lateral spinal flexion (*p* = 0.073) and presence of spondylolisthesis (*p* = 0.080) were predictors just short of significance, whereas presence of active physiotherapy prescription (*p* = 0.044) was a significant predictor for reaching MCID in both HRQOL scores (Table 4).

A review of these patients at 2022 (> 2-years after completion of recruitment) found that additional 2 patients had surgery since the 1-year follow-up of the study period. One patient had deformity correction at 2.9 years after recruitment, while another had the surgery at 1.2 years after recruitment. Hence, our conservative management regimen was successful for 89% (41/46) of the study cohort who presented with adult deformities without neurological deficit.

## Discussion

A lateral flexion range below 25 degrees and absence of comorbidities were each predictive of MCID improvement in ODI. While for SRS-22r, only lateral flexion range was identified as a predictor in multivariate analysis. Lateral flexion range < 25 degrees, presence of spondylolisthesis, and use of active physiotherapy were predictors for achieving MCID in both ODI and SRS-22r. Significant cut-off values for trunk shift > 14 mm, pelvic incidence > 50 degrees, EQ-5D-5L anxiety/depression dimension score > 1, and SRS-22r total score < 3.5 were defined for MCID improvement in SRS-22r score.

Among the radiographic parameters, only a larger trunk shift, a larger pelvic incidence, and presence of spondylolisthesis were identified as predictive factors for successful conservative treatment. These parameters contribute to the body’s capability to achieve an economic sagittal balance [29] and hence deviations are expected to adversely affect the self-image and daily function of patients with scoliosis. Interestingly, coronal Cobb angle and C7-CSVL deviation, the most commonly used radiological parameters defining coronal spinal deformity, were not significant predictors for MCID improvement. However, the power of this research can be relatively weak due to the small sample size. While a similar study by Slobodyanyuk et al [8] could not identify any radiographic predictors, Liu et al [6] suggested that a smaller Cobb angle was a predictor for > 1 MCID improvement in pain or activity domains for the SRS-22r. This discrepancy in treatment outcome with the presence of coronal imbalance may be explained by an uncompensated fractional curve in the lumbosacral region. Additionally, Souder et al [30] mentioned that increased trunk shift alone is a significant predictor for surgery despite a relatively small major curve angle, especially in younger patients due to its impact on external appearance. Addressing cosmetic issues during conservative management of patients may help reduce the need for surgery among patients with relatively small Cobb angles.

Patients with spondylolisthesis are more likely to achieve MCID in both ODI and SRS-22r simultaneously. This suggests the possibility that spondylolisthesis may be more receptive to routine non-operative treatments, as suggested by Lindgren, who prescribed a combination of stretching, coordination and strengthening exercises to symptomatic patients with spondylolisthesis, after which most had become asymptomatic [31]. Meta-analysis also reported that over 80% of patients with spondylolisthesis responded well to non-operative treatment [32].

Patients reaching MCID in ODI and SRS-22r showed less comorbidities (diabetes mellitus type I and II, hypertension, ischemic heart disease, osteoarthritis, cancer). With an aging population, management of age-related comorbidities, has been repeatedly emphasized as one of the key considerations when managing elderly patients with adult degenerative spinal disease [33, 34]. Any possible improvement of spinal condition by non-operative treatment may be masked by exacerbation of other comorbidities, leading to an overall decline in HRQoL.

Smaller range of lateral spinal flexion at baseline was a significant predictor for reaching MCID in ODI. Restriction in lateral spinal flexion had been reported as one of the predictors for development of low back pain, as lateral flexion facilitates force absorption in the spine [35]. While lateral flexion is not as commonly used as anterior flexion and posterior extension in daily life, it is an important indicator of spine mobility, and its reduction is often a sign of lumbar spinal deformity, such as degenerative disc disease [36]. It may be possible to reduce the occurrence and exacerbation of spinal pathologies if patients’ lumbar range of motion is improved [35]. An increase in lateral spinal flexion range during treatment is associated with better back pain relief and improvement in physical performance [37, 38]. One possible mechanism for improvement is the load-induced interstitial fluid flow in intervertebral disc, leading to improved disc nutrition [39]. Hence, targeting this range of motion in conservative treatment may largely improve the symptomatology.

Baseline EQ-5D-5L anxiety/depression dimension score > 1 (indicating more anxiety/decompression) and SRS-22r total score below 3.5 (indicating lower HRQoL) were predictive factors for MCID improvement in SRS-22r. Despite common belief that non-operative treatment is best-suited for patients with minimal degree of deformity and disability, some of the predictors identified in the study included these poorer HRQoL scores which are indicative of a more severe functional impairment. Liu et al [6] noted that patients with greater baseline pain are more likely to have significant improvement in pain, while Slobodyanyuk et al [8] suggested that patients with worse scores in SRS-22 domains of pain, activity, appearance and mental were more likely to achieve > 1 MCID improvement in the corresponding domains. Similar findings are also seen in studies on surgical treatment, as patients with the worst baseline scores and disabilities were more likely to improve substantially following surgery, since their severe disabilities also mean ample room for clinical improvement. This is consistent with our findings that patients with poorer baseline HRQOL scores and radiological parameters were more likely to achieve MCID improvement. It should be noted that patients with lower disability at baseline also showed lower post-treatment disability. Significant correlation between baseline and final scores in ODI, SRS-22r total, pain, and self-image also indicate that patients with better initial health are more likely to have better post-treatment status.

Overall, there was no statistically significant change in any of the HRQOL domains from baseline to 1-year follow-up. Despite physiotherapy being a significant predictor for reaching MCID in both ODI and SRS-22r, there was no statistically significant difference in HRQOL change (*p* = 0.223 for ODI, *p* = 0.734 for SRS-22r change) between those who did or did not attend physiotherapy. While patients may have deteriorated without the physiotherapy, the results question the value and cost-effectiveness of non-operative treatments for adult spinal deformity patients if prescribed indiscriminately. Nevertheless, it was identified that a subset of patient with certain characteristics can achieve > 1 MCID improvement. Correctly identifying this subset of patient among all who sought medical advice will be integral to successful non-operative care of patients.

Some limitations should be reported. As a prospective observational study, patients were recruited for data collection, and no adjustment or reallocation was made to cohorts receiving different treatments, this prevents a fair comparison between treatment protocols. However, because of this, there is a relatively smaller sample size within the study period. This may mask some statistically less prominent predictors and also highlight how this population may not often resort to surgery. Since there were only 3 patients who switched to surgical treatment, the analysis for predictors of avoidance of surgery with non-operative care was not feasible. Nevertheless, certain predictors were identified for MCID improvement which is the defining parameter for satisfactory gains in HRQoL measures. In addition, this study did not evaluate factors that predict treatment effectiveness in terms of prevention of deterioration, which is a major goal of conservative treatment. To our knowledge, there is no established MCID threshold for clinical deterioration in patients with adult spinal deformity.

## Conclusion

This prospective study identified several clinical and radiological parameters for successful nonoperative treatment for adult spinal deformity. Patients with lateral flexion range < 25 degrees, trunk shift > 14 mm, pelvic incidence > 50 degrees, EQ-5D-5L anxiety/decompression dimension score > 1, SRS-22r total score > 3.5, absence of comorbidities and presence of spondylolisthesis carry a higher likelihood of achieving MCID improvement in HRQoL scores.

## Data Availability

The datasets used and/or analyzed during the current study are available from the corresponding author on reasonable request. They are not publicly available as they are currently part of a prospective cohort that will be used for future analyses.

## Abbreviations

CSVL: Central sacral vertical line
EQ-5D-5L: EuroQol 5-dimension 5-level
HRQoL: Health-related quality of life
MCID: Minimal clinically improved difference
ODI: Oswestry disability index
ROC: Receiver operating characteristic
TUG: Timed up and go test
SRS-22r: refined 22-item Scoliosis Research Society
SVA: Sagittal vertical axis
